# A pilot and feasibility study of a cognitive behavioural therapy-based anxiety prevention programme for junior high school students in Japan: a quasi-experimental study

**DOI:** 10.1186/s13034-019-0300-5

**Published:** 2019-10-31

**Authors:** Ikuyo Ohira, Yuko Urao, Yasunori Sato, Toshiyuki Ohtani, Eiji Shimizu

**Affiliations:** 1 United Graduate School of Child Development, Osaka University, Kanazawa University, Hamamatsu University School of Medicine, Chiba University and University of Fukui, 2-2 Yamadaoka, Suita-shi, Osaka, 565-0871 Japan; 2 Research Center for Child Mental Development, Chiba University Graduate School of Medicine, 1-8-1 Inohana, Chuo-ku, Chiba-shi, Chiba, 260-8670 Japan; 3 Department of Preventive Medicine and Public Health, Keio University School of Medicine, 35 Shinanomachi, Shinjuku-ku, Tokyo, 160-8582 Japan; 4 Safety and Health Organization, Chiba University, 1-33 Yayoi-cho, Chiba-shi, Chiba, 260-8670 Japan; 5 Department of Cognitive Behavioural Physiology, Chiba University Graduate School of Medicine, 1-8-1 Inohana, Chuo-ku, Chiba-shi, Chiba, 260-8670 Japan

**Keywords:** Anxiety, Prevention, Cognitive behavioural therapy, Junior high school, Universal, Japan

## Abstract

**Background:**

There is a good deal of evidence that cognitive behavioural therapy is effective for children and adolescents with anxiety-related problems. In Japan, an anxiety prevention programme based on cognitive behavioural therapy called ‘Journey of the Brave’ has been developed, and it has been demonstrated to be effective for elementary school students (aged 10–11 years). The purpose of this study was to have classroom teachers deliver the programme to junior high school students (aged 12–13 years) and to test the feasibility and efficacy of the programme in this setting.

**Methods:**

This study was a prospective observational study and was approved by the Chiba University Review Board. An intervention group consisting of six classes of students in their first year of junior high school at two different schools (*n* = 149; 81 boys, 68 girls) received seven 50-min programme sessions. Participants in the control group were recruited from four classes of students in their second year of junior high school at one school (*n* = 89; 51 boys, 38 girls). All participants completed the Spence Children’s Anxiety Scale at pre-test, post-test, and 2–3 month follow-up. Statistical analysis was conducted using a mixed-effects model for repeated measures model.

**Results:**

Mean total anxiety scores indicated a non-significant decrease at the 2–3 month follow-up for the intervention group compared to the control group. The group differences on the SCAS from baseline to post-test was − .71 (95% CI − 2.48 to 1.06, *p* = .43), and the 2–3 month follow-up was − .49 (95% CI − 2.60 to 1.61, *p* = .64).

**Conclusions:**

In this pilot study, implementation of the programme confirmed the partial feasibility of the programme but did not elicit a significant reduction in anxiety scores. In addition, there are several methodological limitations to this study. In the future, we propose to test the feasibility and efficacy of the programme with the required sample size and by comparing groups with equal characteristics as well as by carrying out additional follow-up assessments.

*Trial registration* UMIN000032517.

## Background

Anxiety disorders are one of the most common types of psychiatric disorder [1], with the lifetime prevalence of any anxiety disorder in children and adolescents ranging from 8.8 to 31.9%. The average age of onset for anxiety disorders is 11 years [2], and such disorders are likely to become chronic [3]. It is believed that anxiety often leads to depression; for example, according to the results of a follow-up survey conducted 10 years after a longitudinal study of anxiety and depressive disorders in adolescents, anxiety disorder in adolescents is associated with a relatively high risk of anxiety or depressive disorders in adulthood [4]. In Japan, a study examining the relationship between anxiety and depression among junior high school students found a significant longitudinal relationship between these disorders after 3 months [5]. Thus, the symptoms of many anxiety disorders are chronic, and anxiety has been found to increase the risk of depression and other psychiatric disorders.

Anxiety disorders in children and adolescents interfere with their school life; for example, it has been shown that they result in school refusal and a decline in academic performance [6, 7]. The results of a previous study of school refusal among adolescents indicate that this is often caused by anxiety disorders. Anxiety disorders are observed in approximately 50% of individuals in representative samples of clinic-referred youth exhibiting school refusal [8]. Particularly, in Japan, it has been pointed out that the problem of school refusal is strongly related to anxiety. According to a survey conducted by the Ministry of Education, Culture, Sports, Science and Technology in 2017 [9], the number of school refusals among elementary and junior high school students is more than 140,000, representing a higher proportion of the population than previously seen. It has also been reported that the proportion of students with tendencies to anxiety is up to 33.2%, which is a contributing factor to this state of affairs.

The relationship between anxiety and academic achievement has also been studied. In recent years, the relationship between developmental disorders and school maladaptation has attracted much attention; however, there is a possibility that children and adolescents who have difficulty concentrating or paying attention in school as a result of anxiety problems tend to be misdiagnosed as having attention deficit hyperactivity disorder (ADHD) [10]. Furthermore, it has also been pointed out that children diagnosed with a learning disability or ADHD include those who show poor performance because of high anxiety [11]. As mentioned above, it has been shown that anxiety problems among children and adolescents cause maladaptation to school life, and in turn, this maladaptation may later become a factor in other comorbidities, such as anxiety disorders and depression. Therefore, it is important to provide early preventive interventions for children and adolescents with the aim of preventing anxiety problems.

Although support during adolescence is regarded as important, many adolescents who have anxiety do not receive appropriate support [12]. In addition, in many cases, it takes a considerable amount of time for patients to begin receiving treatment after the onset of a disorder [13]. A lack of knowledge about mental health and the stigma attached to mental health problems are considered factors in this delay in obtaining support; acquiring accurate knowledge about mental health in school classes is effective in preventing such delays [14]. Puberty, also referred to as ‘the second birth’ [15], is regarded as a developmental stage during which individuals are particularly sensitive to others’ evaluations of them, in addition to being a period of remarkable mental and physical development; thus, it is also a period during which various emotional and behavioural problems become more likely [16]. It is reported that adolescents may present with more severe forms of anxiety-based school refusal than do younger children, and in adolescents, this is also more frequently associated with depressive disorders [17]. It is clear that the presence of an anxiety disorder in this age group is a high-risk factor for serious mental health problems, and support must be offered to children and adolescents in an effective and accessible form [18].

Cognitive behavioural therapy (CBT) is an evidence-based psychological treatment method that can alleviate and improve emotional problems such as anxiety and depression. School-based treatment programmes based on CBT for anxiety, depression, and other problems in children have been found to be effective in randomised controlled trials [19]. Furthermore, attention has been paid to a CBT-based approach to anxiety prevention, which has been found to be effective when delivered in schools [20].

Preventive interventions for mental disorders are classified into three levels by the Institute of Medicine (IOM): (1) universal interventions, (2) selective interventions, and (3) indicated interventions [21]. Universal interventions target the whole population, including those who have no symptoms of the relevant disorder. Selective interventions target individuals or groups who are at a higher than average risk. Lastly, indicated interventions target individuals or groups who are already experiencing a low-to-moderate level of symptoms, and therefore, are at a high risk of developing the disorder in the future.

For students, school is a natural and familiar place, and the implementation of a universal prevention programme in schools enables students to receive treatment more easily in terms of time, place, and cost, and may provide them with skills and strategies that help prevent or delay the onset of mental disorders [22–24]. Therefore, it can be argued that it is of great importance to implement a universal prevention programme to prevent future anxiety disorders and to reduce the risk of comorbidity, even in children without particular symptoms or signs at the time of the intervention. Although the delivery of a mental health programme in school by class teachers has an especially low cost, which makes continued implementation of such a programme possible, the results of a randomised controlled trial of a universal prevention programme for anxiety in school did not demonstrate the effectiveness of the teacher’s conduct [25]; however, other randomised controlled trials have found that in the trauma-focused group intervention ‘Mein Weg’ for young refugees, lay counsellors’ conduct in a psychosocial intervention was effective [26, 27]. As mentioned above, numerous benefits of implementing the programme at the school exist, and we believe that it would be beneficial for the teacher to participate in this programme.

‘Friends’ is a universal programme aimed at preventing childhood and adolescent anxiety [28]. This programme has been shown to be effective in adolescents (aged 14–16 years), although the effect of the intervention on this group is small compared to its effect on younger children (aged 9–10 years) [29]. However, implementation of the ‘Friends’ programme in Japan did not lead to a significant reduction in total anxiety scores [30]. Therefore, it might be effective to apply a programme developed according to the social and cultural background of Japan. In Japan, a CBT-based anxiety prevention programme called ‘Journey of the Brave’ that can be implemented as part of the Japanese school curriculum has been developed [31]. In a previous study on fifth year elementary school students (intervention group *n* = 41, control group *n* = 31), trained health facilitators (with graduate school training in CBT) conducted 10 sessions in the classroom as a school lesson [32]. The mean anxiety score on the SCAS for the intervention group had significantly reduced at both post intervention and the 3-month follow-up compared to the control group.

Although research into this topic targeting junior high school students have not so far been conducted in Japan, we believe that it is important to tackle potential mental health problems in junior high school students, given that as described above, they may face an ‘adolescent crisis’ at a mentally and physically sensitive stage of their life.

Furthermore, in Japan, the first year of junior high school is also the year in which students experience major changes in their educational environment. First, as multiple elementary schools feed into each junior high school, the school and its classes are larger in size, and students experience major changes in their peer relationships. Second, elementary school and junior high school differ greatly in terms of the student–teacher relationship. In elementary school, the so-called ‘home room teacher’ system is applied, while the junior high school follows the curriculum management system (different areas of the curriculum are taught by specialised teachers). Finally, the number of subjects and the degree of learning difficulty increase. In addition to experiencing such environmental changes, researchers have pointed out that junior high school students are also approaching a sensitive stage of adolescence, during which various psychological and behavioural problems may come to the surface [33].

The ‘Journey of the Brave’ programme was originally developed for children in the fourth to sixth year of elementary school. However, because the programme was designed based on evidence-based CBT theory and tackles ways to cope with anxiety in interpersonal relationships, it seems likely that this programme could be adapted for use among junior high school students. Therefore, in this pilot study, we aimed to implement this programme among junior high school students, with the classroom teacher acting as a facilitator, and to test its feasibility and efficacy with the aim of preventing anxiety problems.

## Methods

### Study design and participants

This study was conducted in collaboration with Chiba University and Kodomo Minna Project (‘Project for all the children’). This is a project in which ten universities collaborated and conducted a research, commissioned by the Ministry of Education, Culture, Sports, Science and Technology, for the purpose of improving school refusal and bullying, which are major issues in Japanese schools.

This is part of a research project on students from elementary to high school. In this study, data on junior high school students were collected and analysed. The Ministry of Education, Culture, Sports, Science and Technology recruited schools to participate in this programme. The Board of Education of a prefecture located in the western part of Japan applied to participate, and students in their first year of junior high school were selected to participate in the programme. Although it would have been desirable methodologically to recruit a control group from students in the same year, the Board of Education made a firm request for all first-year students in the participating schools to receive the programme at the same time; therefore, students in their second year of junior high school were recruited for the control group.

This was a universal quasi-experimental study with an intervention and a control group. The participants in the study were 472 students in their first or second year of junior high school (aged 12–14 years), attending three public junior high schools in a single prefecture in Japan. Intervention group participants received the anxiety prevention programme, and control group participants received no prevention programme.

In addition, the ‘Journey of the Brave’ programme was conducted as part of regular classes in schools. This study was a prospective observational study that collected and analysed students’ anxiety scores before and after the programme. It was approved by the Chiba University Review Board. In this study, consent was obtained in the form of an opt-out. Parents were given an informational letter about the study, and they could provide opt-out consent to exclude their child from participation. In addition, at the time of the survey, teachers distributed a written assent form for the students, for students to provide their assent to participate.

### Prevention programme: ‘Journey of the Brave’

Table 1 provides a summary of the ‘Journey of the Brave’ programme. This is a programme developed with consideration for the psychological characteristics of children and adolescents and for the social and cultural background of Japan, with the following three representative features [31]. First, this programme specialises in the prevention of anxiety-related problems, to help children and adolescents understand the purpose of the programme and engage in effective learning. Second, in order to enable children and adolescents to enjoy the programme, likeable characters are presented in a story format. Third, group work is intentionally avoided in favour of emphasising an individual work format because of the psychological characteristics of Japanese adolescents. It has been pointed out that compared to individuals in Western countries, Japanese individuals tend to be more influenced by the way they are perceived by others [34]. Adolescents tend to feel more anxious about the relationships within the same age group [35], and it is necessary to consider that there may be some students with high anxiety in the class.

**Table 1:** Contents of ‘Journey of the Brave’ by session

Session at the junior high school	Original session	Content of ‘Journey of the Brave’
1	1	Understanding of the four basic feelings
2	2	Monitoring the feelings of anxiety and setting goals
	3	Body reactions and relaxation
3	4	Anxiety-level stages and hierarchical exposure
4	5	Anxiety cognition model
	6	Identifying cognitive distortions and coping with rumination
5	7	Cognitive restructuring when anxious
6	8	Assertiveness skills to reduce social stress
7	9	Review
	10	Summary

This programme consists of ten 45-min sessions; the content is taught according to a workbook and a teacher’s manual. The first half of the programme is dedicated to the development of ‘anxiety hierarchy’ and the experience of gradual exposure, while the second half mainly concerns cognitive restructuring. More precisely, after psychological education on anxious feelings (i.e., the notion that anxiety is a natural feeling that everybody has and plays an important role in protecting you from danger, but if excessive anxiety persists, it might lead to disturbances in life, etc.), each student is encouraged to establish his or her own goal for the programme, such as giving a presentation in front of all the students, an important test, and so on. In stage 3, relaxation skills such as breathing methods and muscle relaxation are taught. In stage 4, students develop a table of their ‘anxiety hierarchy’, consisting of 7 steps that will allow them to reach the goal set in stage 2. Stages 5, 6, and 7 encompass the process of gradually learning about the cognitive model (the relationship between cognition, behaviour, emotion, and bodily responses) as well as cognitive restructuring. At the same time, gradual exposure homework is given to address higher levels of anxiety in accordance with the anxiety stairs table developed in stage 4. Assertion skills to reduce interpersonal anxiety are taught in stage 8; stage 9 consists of an overall review session; and stage 10 involves a summary and graduation ceremony. In the workbook used by the students, realistic examples of many anxiety-provoking moments in their daily lives are provided so that they can deepen their understanding of anxious feelings and CBT.

### Procedure

The original ‘Journey of the Brave’ programme consisted of 10 sessions (administered once per week, each lasting 45 min). As this study conducted the programme in junior high schools, the research group elected to reduce the number of sessions in view of the fact that the length of class time was 5 min longer than in elementary school, and that junior high school students should be able learn more quickly. In addition, since the curriculum of the regular classes for the year has already been determined, the Board of Education requested that the number of classes be reduced to seven that were administered about once per week and lasting 50 min.

In this programme, the content of each session was based on CBT theory (Table 1), but the relaxation method (Stage 3) could be shortened as it was addressed in health class, and Stages 2 and 3 were consolidated into one session. The remaining content was implemented within the 7 class hours. As Stages 5 and 6 as well as Stages 9 and 10 had little individual work for students, we decided to summarize these in one session.

Additionally, the following three things were addressed as we utilized a group of practitioners who did not have specialized knowledge about CBT to allow them to lead this programme smoothly and effectively. First, we conducted a 6-h workshop, which was a training course. This training course was a free workshop, and participants received a certificate of completion. This workshop consists of lectures on the theory of CBT, role-plays for each session (lasting about 20 min per session), feedback from instructors, and time for questions and answers. Second, we devised a workbook with detailed contents that allowed the students to read and understand it themselves. Third, we had them utilize a teacher’s manual, which was distributed to the teachers. The teacher’s manual was attached with the Q & A and information on how to proceed with the class, which was created based on questions by teachers in past programmes. In addition, after the completion of stage 3, a template for reporting the progress of the class was attached to the teacher’s manual. In the report template, there is a field for comments and consultations for supervision. In addition, if the teachers wanted to have a consultation, they could do so at any time by phone or email during the intervention period. This was described in the manual and shared with the teachers at the workshop.

The preventive interventions were conducted from September to November 2017 in one participating school and from October to December 2017 in the other. In each case, the intervention was delivered by the class teacher, who had taken the ‘Journey of the Brave’ programme instructor training course. In total, the programme was implemented by the class teacher in six classes of two junior high schools.

All sessions were held in the classroom during regular class time. Every session was conducted according to the workbook and the teacher’s manual, and a piece of homework was to be assigned at the end of each session, to be worked on at home and returned by the next session, in order to help students consolidate the content. Students in the control group followed the regular school curriculum. The main assessments were a pre-test (Time 1; baseline), a post-test (Time 2; 2–3 months after baseline), and a follow-up test (Time 3; 2–3 months after the post-test). At each of these time points, self-report questionnaires were distributed to the students by the teacher in charge of each class, and all students (149 in the intervention group and 89 in the control group) completed the questionnaires. The teachers assisted students in this process by reading the questions aloud.

### Measurements

#### Primary outcome measure: Spence Children’s Anxiety Scale

The Spence Children’s Anxiety Scale (SCAS) [36] is a self-report measure of anxiety symptoms designed for children and adolescents. The scale consists of 38 items relating to anxiety symptoms, divided into six subcategories: separation anxiety, social phobia, panic disorder/agoraphobia, generalised anxiety disorder, fear of physical injury, and obsessive–compulsive disorder. Possible item scores range between 0 (*never*) and 3 (*always*), and the maximum possible score is 114. Ishikawa et al. [37] developed a Japanese version of the SCAS with good internal reliability coefficients. According to a previous study, the average SCAS score among 7- to 19-year-old children and adolescents is 18.11 (*SD* = 12.87), and the cut-off point is 35 [38].

#### Secondary outcome measure: Emotion-Regulation Skills Questionnaire

The Emotion-Regulation Skills Questionnaire (ERSQ) [39] is a self-report questionnaire consisting of 27 items. Possible item scores range between 0 (*not at all*) and 4 (*almost always*), and the maximum possible score for the questionnaire is 108. In the original version, successful application of emotion-regulation skills is assessed through the following nine subscales: awareness, sensation, clarity, understanding, modification, acceptance, tolerance, readiness to confront, and compassionate self-support. Fujisato et al. [40] developed a Japanese version of the ERSQ with good internal reliability coefficients. In the Japanese version, items are divided into two subcategories: acceptance and engagement (tolerance, modification, readiness to confront, and acceptance) and awareness and understanding (sensation, awareness, understanding, clarity, and compassionate self-support).

### Programme evaluation form for students

Students were asked to evaluate the programme after completing all seven sessions. An evaluation form was used to measure their acceptance of and satisfaction with the programme. The form comprised the following two sections: (1) the student’s evaluations of the content of the programme (5 items; for example, ‘Do you think that this programme helped you to cope well with your feelings of anxiety?’ with each item scored from 0 = *disagree* to 3 = *agree*; see Additional file 1: Table S1) and (2) the student’s accomplishment of their ‘anxiety hierarchy’ task (scored from 0 = *none* to 3 = *complete*).

### Statistical analysis

For baseline variables, summary statistics are presented in the form of frequencies and proportions for categorical data, and means and SDs for continuous variables.

Analysis of the primary outcome measure consisted of a mixed-effects model for repeated measures (MMRM), with intervention group, time, and the interaction between intervention group and time as fixed effects; an unstructured covariate was used to model the covariance of within-participant variability. MMRM analysis assumes that any missing data occur randomly. Analysis of the secondary outcome measure was performed in the same manner. We also conducted subgroup analysis by comparing the intervention and control groups on their SCAS scores in a high-anxiety subgroup (SCAS score of 35 points or above in the pre-test) and a low-anxiety subgroup (SCAS score below 35 in the pre-test). Subgroup analysis was also performed in the same manner.

Additionally, the responses to the students’ evaluation questionnaires were aggregated. A repeated-measures analysis of variance (ANOVA) was conducted to examine the changes in SCAS scores at each time point according to the students’ responses regarding the extent to which they had accomplished their ‘anxiety hierarchy’ task (0 = none to 3 = complete).

All comparisons were planned and all *p* values reported are two-tailed. A *p* value < .05 was considered to represent statistical significance. All statistical analyses were performed using the SAS software program, version 9.4 (SAS Institute, Cary, NC, U.S.A.), and SPSS Version 24.0 (IBM, Armonk, New York, USA).

## Results

**Fig. 1:**
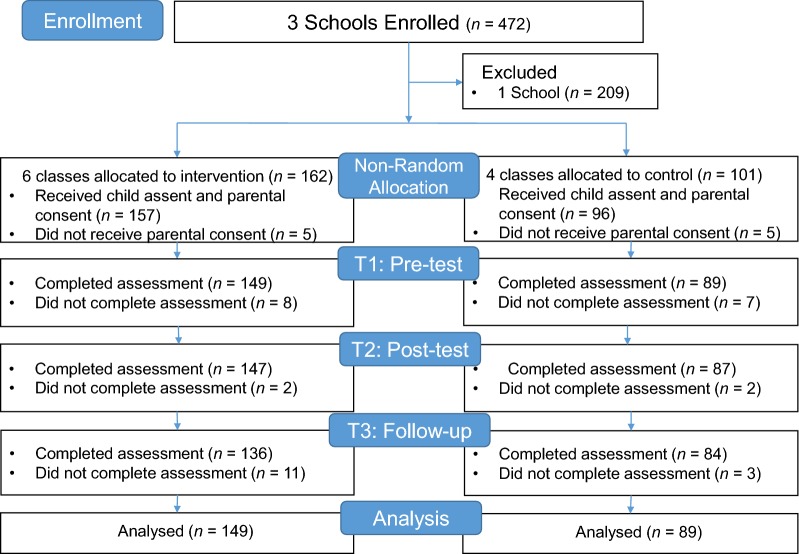
displays the number of students at each time point and sample count of the ITT analysis. *ITT* intention to treat

Three schools agreed to participate in this study, but one was excluded from participation before the baseline assessments because it could not deliver the full programme during the requisite school year. As a result of confirming parental consent and the student’s participation in this research, five parents in intervention group and five parents in control group did not provide consent. All students assented to participate. Thus 253 of 263 eligible students at two junior high schools had valid consent to participate. The intervention group consisted of first-year students (aged 12–13 years) in six classes of two junior high schools. The control group consisted of second-year students (aged 13–14 years) in four classes of one junior high school. The final number of participants entered into the analysis was 149 in the intervention group (81 boys, 68 girls) and 89 in the control group (51 boys, 38 girls; Fig. 1).

Pearson’s correlation coefficient indicated that there was a weak negative correlation between SCAS and ERSQ scores at pre-test, *r* = − .19, *p* < .001. Next, the intervention group and control group were tested for differences in gender ratio at pre-test using the Chi squared test. There was no significant difference (*p* = .66). Finally, *t* tests were conducted to compare the groups at baseline on their pre-test SCAS and ERSQ scores. The intervention group exhibited higher SCAS scores than those of the control group (*p* = .02). However, there were no significant differences in ERSQ scores between the two groups (*p* = .61).

Tables 2, 3, 4 present the results of the MMRM analysis of the intervention and control groups’ SCAS and ERSQ scores at each time point. In the primary analysis of SCAS scores, the estimated mean changes in SCAS score between baseline and follow-up according to the model were − 2.20 (95% CI − 3.49 to − .91) and − 1.70 (95% CI − 3.37 to − .05) for the intervention and control groups, respectively; the difference between groups was − .49 (95% CI − 2.60 to 1.61, *p* = .64; Table 2).

**Table 2:** SCAS scores over time

Test	Intervention group (*n* = 149)	Control group (*n* = 89)	Between group differences for baseline change	*p*
Pre	21.24 (18.88–23.60)	17.40 (14.39–20.42)		
Post	19.21 (16.85–21.56)	17.21(14.20–20.21)	− .71 (− 2.48 to 1.06)	.43
Follow-up	18.86 (16.49–21.23)	15.31 (12.30–18.32)	− .49 (− 2.60 to 1.61)	.64

Estimated mean score on the SCAS at each time point and estimated difference in change between the groups according to a mixed effects model for repeated measures. Scores are presented in the form *M* (95% CI). *SCAS* Spence Children’s Anxiety Scale

**Table 3:** ERSQ scores over time

Test	Intervention group (*n* = 140)	Control group (*n* = 86)	Between group differences for baseline change	*p*
Pre	57.28 (52.89–61.68)	60.95 (55.34–66.55)		
Post	59.52 (55.12–63.92)	60.79 (55.17–66.40)	2.14 (− .52 to 4.80)	.11
Follow-up	59.27(54.85–63.68)	61.64 (56.03–67.25)	1.52 (− 2.10 to 5.14)	.41

Estimated mean score on the ERSQ at each time point and estimated difference in change between the groups according to a mixed effects model for repeated measures. Scores are presented in the form *M* (95% CI). *ERSQ* Emotion-Regulation Skills Questionnaire

**Table 4:** SCAS scores over time: subgroup

Test	Intervention group	Control group	Between group differences for baseline change	*p*
High-anxiety	*n *= 19	*n *= 8		
Pre	51.74 (46.65–56.82)	45.88 (38.04–53.72)		
Post	45.80 (40.65–50.95)	46.88 (39.04–54.72)	− 5.89 (− 13.71 to 1.94)	.13
Follow-up	47.80 (42.47–53.14)	46.75 (38.46–55.05)	− 4.70 (− 13.02 to 3.62)	.26
Low-anxiety	*n *= 130	*n *= 81		
Pre	16.76 (15.05–18.47)	13.58 (11.42–15.74)		
Post	15.28 (13.57–16.98)	12.56 (10.39–14.73)	.03 (− 1.73 to 1.78)	.97
Follow-up	14.67 (12.95–16.40)	11.81 (9.64–13.98)	.09 (− 2.05 to 2.22)	.94

High-anxiety subgroup (SCAS scores ≥ 35), Low-anxiety (SCAS scores < 35) subgroup analysis: estimated mean score on the SCAS at each time point and estimated difference in change between the experimental groups according to a mixed effects model for repeated measures. Scores are presented in the form *M* (95% CI). *SCAS* Spence Children’s Anxiety Scale

In the secondary analysis, the estimated mean changes in ERSQ score between baseline and follow-up according to the model were 2.13 (95% CI − .15 to 4.41) and .61 (95% CI − 2.20 to 3.42) for the intervention and control groups, respectively; the difference between groups was 1.52 (95% CI − 2.10 to 5.14, *p* = .41; Table 3).

In the subgroup analysis of the high-anxiety group (SCAS scores ≥ 35), the estimated mean changes in SCAS score between baseline and follow-up according to the model were − 3.81 (95% CI − 8.25 to .63) and .89 (95% CI − 6.04 to 7.82) for the intervention and control groups, respectively; the difference between groups was − 4.70 (95% CI − 13.02 to 3.62, *p* = .26; Table 4). Additionally, in the subgroup analysis of the low-anxiety group (SCAS scores < 35), the estimated mean changes in SCAS score between baseline and follow-up according to the model were − 1.94 (95% CI − 3.26 to − .62) and − 2.03 (95% CI − 3.70 to − .36) for the intervention and control groups, respectively; the difference between groups was .09 (95% CI − 2.05 to 2.22, *p* = .94; Table 4).

### Students’ programme evaluations

Additional file 1: Table S1 presents the number and percentage of respondents giving each response to each item on the programme efficacy section of the evaluation questionnaire.

According to the repeated-measures ANOVA to examine SCAS scores at each time point based on students’ responses regarding the extent to which they had accomplished their ‘anxiety hierarchy’ task (Table 5), there was a no significant interaction effect between group and time (*p* = .85).

**Table 5:** SCAS scores according to success in accomplishing ‘anxiety hierarchy’ task (*n* = 132)

	‘Anxiety hierarchy’ task accomplishment			
Test	None (*n *= 15)	A little (*n* = 52)	Almost complete (*n* = 56)	Complete (*n* = 9)
Pre	23.87 (18.24)	22.33 (16.80)	19.34 (11.82)	14.33 (11.21)
Post	22.07 (19.27)	20.87 (15.92)	17.04 (11.30)	13.89 (13.82)
Follow-up	23.60 (20.00)	20.06 (15.93)	16.48 (12.88)	13.33 (12.53)

SCAS scores at each time point according to the extent to which students reported that they had succeeded in accomplishing their selected ‘anxiety hierarchy’ exposure task. Scores are presented in the form *M* (*SD*). *SCAS* Spence Children’s Anxiety Scale

## Discussion

In this study, we delivered the universal anxiety prevention programme ‘Journey of the Brave’ to junior high school students in Japan and tested its feasibility and efficacy in reducing anxiety. First, none of the schools dropped out, and all seven sessions were possible within the schools’ curriculum. In addition, the results of the students’ responses in the evaluation questionnaire (Additional file 1: Table S1) showed an overall positive evaluation. Thus, the feasibility of programme implementation in junior high school was partially confirmed. Next, the results indicated that there was no significant difference between the intervention and control groups in terms of change in SCAS scores or ERSQ scores. Furthermore, in a subgroup analysis, the intervention group’s SCAS scores were not significantly reduced in either the high-anxiety group (SCAS scores ≥ 35) or the low-anxiety group (SCAS scores < 35).

In this pilot study, programme implementation did not elicit a clear reduction in student’s anxiety, nor did it clearly show a relationship between anxiety and emotional regulation skills. However, there are several factors to consider as possible reasons for the lack of reduction in students’ anxiety in the intervention group.

### Student’s anxiety

The results for anxiety are in contrast to those of the original study of this preventive intervention on elementary school students [32], in which a significant reduction in the anxiety scores of the intervention group was observed. We consider two possible reasons for the absence of a significant reduction in anxiety scores in the present study. The first reason is that this programme was facilitated by classroom teachers with limited expertise for CBT, whereas in the original study, the programme was conducted by trained health facilitators. The second is that the environmental surroundings of junior high school students differ greatly from those of elementary school students, and the former group are at a sensitive and difficult developmental stage compared to elementary school students.

In the previous study with elementary school students, the programme was delivered mainly by trained health facilitators, but in the present study, the intervention was delivered by teachers. In a UK-based study of the effect of universal anxiety prevention programmes in schools, it has been reported that intervention by trained health facilitators is effective, but that teacher-led intervention may not be effective [25]. In this study, when classroom teachers acting as facilitators were asked about the amount of homework assigned, they reported that homework assignment and review was not practiced regularly at the two schools. Homework is considered one of the most important therapeutic components of CBT [41]. In CBT, the ultimate goal is for clients to be able to exercise control over their own emotions and behaviours, and the practice provided by homework is useful in establishing knowledge and skills, making use of them in daily life (generalisation), and improving self-efficacy. Previous studies in which this programme has been implemented have also shown that ongoing provision and review of homework helps students to consolidate their knowledge and change their behaviour [32]. Since it can be presumed that the facilitator’s level of expertise in CBT is particularly influential with regard to homework assignment and students’ accomplishments with gradual exposure (reported in the present study as part of the students’ programme evaluation questionnaires), it is possible that differences in the expertise of facilitators may have caused the disparity in effects between the original and the present study. In mental health interventions delivered by lay counsellors, supervision has been shown to be important in managing programme fidelity [42, 43]; therefore, it will help the classes progress more effectively if the supervision of the teachers who are leading the sessions can be enriched. In this study, there were no telephone or email consultation requests from the teachers. In addition, in the report template, the teacher reported the completion of stage 3 and the future schedule of the class at the midpoint of this programme. There was a section where comments and consultations from teachers were entered into this report template, but there were only comments on the programme and impressions about the class overall, and no records of consultations. Therefore, for supervision, it will be necessary to improve the report format so that teachers can easily complete assignments and consultations. Furthermore, in future implementation, in addition to using the report template, it will be important to set a time for conducting supervision sessions in advance.

In addition, the workbook used in this programme seems to be appropriate, because it deals with themes that are likely to present issues during adolescence, such as anxiety in interpersonal relationships, but it is possible that the content might not have been suitable for the developmental stage of junior high school students. Feedback from teachers who had been involved in delivering this programme was collected at the end of the intervention, and some teachers mentioned that ‘the illustrations may be too childish for the students’ and ‘some examples of anxiety scenarios don’t match the students’ level of development’. We propose that a future task should be to improve the content of the workbook so that it matches the developmental stage of junior high school students.

Furthermore, in the present study, the number of sessions was reduced from 10 to seven in view of the fact that junior high school students have a higher level of understanding than elementary school students. However, a meta-analysis of research on universal school-based preventive interventions [44] shows that the greater the number of sessions, the larger the effect; thus, it is probable that the negative outcome in the present study may be partially attributable to this reduction of the number of sessions.

We believe that the factors discussed above greatly influenced the students’ motivation for learning during this programme. Therefore, it will be necessary to revise the contents of the programme further, based on the developmental stage of junior high school students and taking into account the evaluations provided by participants in the programme, and to deliver the full 10 sessions in future administrations of the preventive intervention.

A final point to consider is that, in general, it is desirable for participants in both the intervention and control groups to have comparable scores on the outcome measure at baseline; however, in this study, the anxiety scale (SCAS) scores significantly differed between the groups. The participants in this study were recruited from the first year (intervention group) and second year (control group) of junior high school. The first year of junior high school in Japan is a year in which students experience major changes in their educational environment. Research has reported that school refusal and the number of students whose study motivation declines is increasing rapidly [45]. It is estimated that the first year of junior high school is a time when anxiety greatly increases compared to other grades, and the difference between the groups in this study is possibly attributable to the fact that the groups were drawn from different academic years. Additionally, the small number of participants in this study might have influenced this result. The results of the original study (2018) revealed that the smaller the number of participants, the greater the difference in baseline scores between the intervention and control groups. In the future, we plan to verify the efficacy of the programme by recruiting an appropriate number of participants from the same academic year and who have comparable mean total scores on the anxiety scale (SCAS).

### Students’ programme evaluations

Based on the results of the questionnaire items in which students were asked to evaluate the efficacy of the programme (item 3: ‘Do you think that this programme helped you to cope well with your feelings of anxiety?’ and item 5: ‘Do you think that what you learned in this programme will be useful in the future?’), more than 70–80% of the students answered in the affirmative. One of the advantages of implementing a universal prevention programme in schools is the prevention of potential future deterioration of the mental health of students who do not present any symptoms or signs at the time of the programme, and the reduction in the risk of other comorbidities. Although no significant reduction in participants’ SCAS scores was observed on this occasion, we conclude that the delivery of this programme is useful in allowing participants to acquire knowledge and skills regarding how to manage their anxiety, and these techniques can be used to exercise control of their own emotions and behaviours in their future lives. By implementing this universal intervention programme for anxiety prevention in schools, students might acquire the knowledge and skills based on CBT and apply them to prevent mental health deterioration in the future. Therefore, longitudinal studies must be conducted to verify the long-term efficacy of universal preventive interventions [46]; doing so for the programme implemented here, through a follow-up assessment, is a future task.

Furthermore, the results indicated that there was no significant difference in SCAS scores at each time based on students’ responses regarding the extent to which they had accomplished their ‘anxiety hierarchy’ task (0 = none to 3 = complete). However, looking at the change in the score at each stage, we found that the students who reported positive progress in their responses to the item on the extent to which they were able to accomplish their anxiety hierarchy gradual exposure task also exhibited a decrease in SCAS scores at the post-test and follow-up test. In contrast, the scores of students who reported that they had not been able to complete any of the steps toward their task were reduced in the post-test, but subsequently increased again in the follow-up test.

As a second point, when examining total scores in the pre-test, we noticed that the higher the participant’s anxiety score, the lesser the extent to which they were able to accomplish their anxiety hierarchy task. The results of many tests of CBT treatments for anxiety problems in children and adolescents have shown that success with exposure therapy is important to alleviate anxiety [47], but the present study indicated that participants’ degree of exposure achievement was lower among students with higher anxiety scores. Therefore, it is conceivable that students with higher anxiety scores may not have been able to set feasible targets that matched their anxiety level (meaning that it was difficult for them to accomplish the exposure task in their daily lives). In the future, it may be necessary to improve the programme workbook, especially in relation to how to set a reasonable goal so that students can select achievable targets that match their individual capacities in class. Assistance for students with high anxiety who experience difficulty with gradual exposure will also lead to the provision of early intervention and support at school, which will be very helpful to such students.

### Limitations and future prospects

There were several methodological problems and limitations with the present study, as follows. First, because this was a pilot study, the number of participants may have been insufficient. The study enabled the calculation of sample size to detect clinically significant differences in outcome measures. Using the PS Power and Sample Size Calculator Software version 3.1.2 with α equivalent to .05 and power (1−β) of .80, the required sample size for this type of research was found to be 200 participants each for the intervention and control groups [48]. Additionally, in this study, the anxiety scale (SCAS) scores differed significantly between the intervention and control groups, possibly due to differences in grade between the students in these groups. In the future, we aim to verify the efficacy of the programme by recruiting intervention and control groups with an appropriate number of participants from the same academic year.

Next, according to systematic reviews and meta-analyses of school-based anxiety and depression prevention programmes, the effect size of such preventive programmes is small, but it has been indicated that, even with a small effect size, there is a possibility that it can be useful for preventing the onset of these disorders in youth [19]. Additionally, research reports that young people (aged 7–14 years) with anxiety commonly worry about how others perceive them, and thus tend to give socially desirable responses instead of providing valid self-report [49]. In the future, in order to evaluate the effects of preventive programmes, it will be necessary not only to evaluate efficacy using questionnaires (i.e., self-report), but also to design a long-term study in which a follow-up study of participants’ changes in anxiety score and the number of school refusals is conducted.

## Conclusions

Following the delivery by classroom teachers of the universal anxiety prevention programme ‘Journey of the Brave’ for junior high school students in Japan, the feasibility of the programme implementation in junior high school was partially confirmed. However, there was no significant reduction in anxiety scores such as observed following implementation of the same programme in elementary schools. This pilot study represented the first attempt to have classroom teachers deliver this programme and to use the programme with junior high school students. Going forward, in consideration of the results and of the nature of junior high school classes, we intend to improve the efficacy of the programme for this age group by modifying the workbook and number of session as well as by providing more detailed and structured teacher supervision. In addition, as there were several limitations to the design of this study, it will be necessary to test the feasibility and efficacy of the programme with required sample size and equalizing the members of the group. Finally, we need to verify the programme’s preventive efficacy longitudinally by carrying out additional follow-up assessments.

## Supplementary information

**Additional file 1:** Table S1. Responses to students’ evaluation questionnaire (*n* = 146). The number and percentage of respondents giving each response to each item on the programme efficacy section of the evaluation questionnaire.

## Data Availability

The dataset used and analysed during the current study is available from the corresponding author on reasonable request.

## Abbreviations

CBT: cognitive behavioural therapy
SCAS: Spence Children’s Anxiety Scale
ERSQ: Emotion-Regulation Skills Questionnaire
MMRM: mixed-effects model for repeated measures
