# Supplementary material for: A pilot and feasibility study of a cognitive behavioural therapy-based anxiety prevention programme for junior high school students in Japan: a quasi-experimental study
Source: Child Adolesc Psychiatry Ment Health. 2019 Oct 31;13:40. doi: 10.1186/s13034-019-0300-5 (PMC6824127; doi:10.1186/s13034-019-0300-5)
Supplement: Supplementary file 1 — Additional file 1: Table S1. Responses to students’ evaluation questionnaire (n = 146). The number and percentage of respondents giving each response to each item on the programme efficacy section of the evaluation questionnaire. [file 13034_2019_300_MOESM1_ESM.docx]

**Table S1. Responses to students’ evaluation questionnaire (*n* = 146)**

|  |  | *n* (%) | | | | | | | |  |
| --- | --- | --- | --- | --- | --- | --- | --- | --- | --- | --- |
|  | Item | Disagree | | Somewhat disagree | | Somewhat agree | | Agree | | Total (*n*) |
| 1. | Did you have a positive experience taking the ‘Journey of the Brave’ classes? | 2 | (1.4%) | 14 | (9.6%） | 86 | (58.9%) | 44 | (30.1%) | 146 |
| 2. | Was it easy for you to understand the programme ? | 3 | (2.1%) | 17 | (11.6%) | 69 | (47.3%) | 57 | (39.0%) | 146 |
| 3. | Do you think that this programme helped you to cope well with your feelings of anxiety? | 10 | (6.8%) | 30 | (20.5%) | 77 | (52.7%) | 29 | (19.9%) | 146 |
| 4. | Do you want to take classes like the ‘Journey of the Brave’ programme again? | 25 | (17.1%) | 38 | (26.0%) | 73 | (50.0%) | 10 | (6.8%) | 146 |
| 5. | Do you think that what you learned in this programme will be useful in the future? | 10 | (6.9%) | 16 | (11.0%) | 70 | (48.3%) | 49 | (33.8%) | 145 |
